# Scrutinizing the cut-off for “pathological” meniscal body extrusion on knee MRI

**DOI:** 10.1007/s00330-018-5914-0

**Published:** 2019-01-10

**Authors:** F. Svensson, D. T. Felson, A. Turkiewicz, A. Guermazi, F. W. Roemer, P. Neuman, M. Englund

**Affiliations:** 1 Faculty of Medicine, Department of Clinical Sciences Lund, Orthopedics, Clinical Epidemiology Unit, Lund University, Lund, Sweden; 2 Clinical Epidemiology Research & Training Unit, Boston University School of Medicine, Boston, MA USA; 3 Department of Radiology, Boston University School of Medicine, Boston, MA USA; 4 Department of Radiology, University of Erlangen-Nuremberg, Erlangen, Germany

**Keywords:** Meniscus, Osteoarthritis, Cartilage, Magnetic resonance imaging, Knee joint

## Abstract

**Objectives:**

Medial meniscal body extrusion ≥ 3 mm on MRI is often considered “pathologic.” The aims of this study were to (1) assess the adequacy of 3 mm as cut-off for “pathological” extrusion and (2) find an optimal cut-off for meniscal extrusion cross-sectionally associated with radiographic knee osteoarthritis, bone marrow lesions (BMLs), and cartilage damage.

**Methods:**

Nine hundred fifty-eight persons, aged 50–90 years from Framingham, MA, USA, had readable 1.5 T MRI scans of the right knee for meniscal body extrusion (measured in mm). BMLs and cartilage damage were read using the whole organ magnetic resonance imaging score (WORMS). Knee X-rays were read according to the Kellgren and Lawrence (KL) scale. We evaluated the performance of the 3-mm cut-off with respect to the three outcomes and estimated a new cut-off maximizing the sum of sensitivity and specificity.

**Results:**

The study persons had mean age of 62.2 years, 57.0% were women and the mean body mass index was 28.5 kg/m^2^. Knees with radiographic osteoarthritis, BMLs, and cartilage damage had overall more meniscal extrusion than knees without. The 3-mm cut-off had moderate sensitivity and low specificity for all three outcomes (sensitivity between 0.68 [95% CI 0.63–0.73] and 0.81 [0.73–0.87], specificity between 0.49 [0.45–0.52] and 0.54 [0.49–0.58]). Using 4 mm maximized the sum of sensitivity and specificity and improved the percentage of correctly classified subjects (from between 54 and 61% to between 64 and 79%).

**Conclusions:**

The 4-mm cut-off may be used as an alternative cut-off for denoting pathological meniscal extrusion.

**Key Points:**

• *Medial meniscal body extrusion is strongly associated with osteoarthritis.*

• *The 3*-*mm cut-off for medial meniscal body extrusion has high sensitivity but low specificity with respect to bone marrow lesions, cartilage damage, and radiographic osteoarthritis.*

• *The 4*-*mm cut-off maximizes the sensitivity and specificity with respect to all three osteoarthritis features.*

## Introduction

The term meniscal extrusion is often used when the peripheral border of the meniscus is substantially located outside the knee joint margin. Meniscal extrusion has been associated with meniscal tears, meniscal degeneration, and the presence of knee osteoarthritis (OA) [1–20]. We have previously reported that the mean medial meniscal body extrusion in the general population of middle-aged and elderly persons without radiographic knee OA was 3 mm. Certain semi-quantitative magnetic resonance imaging (MRI) OA scoring systems use 2 mm as the recommended starting point to denote the presence of meniscal body extrusion. The Boston-Leeds Osteoarthritis Knee Score (BLOKS) uses a four-point scale (0, < 2; 1, 2–2.9 mm; 2: 3–4.9 mm; 3, > 5 mm extruded) [21–23]. The MRI Osteoarthritis Knee Score (MOAKS) uses the same classification for medial and lateral extrusion [24]. The Whole Organ MR Score (WORMS) initially did not define meniscal extrusion, but then a simpler scale was later added in modifications of the system (0: absent, 1: ≤ 50% extruded, 2: >50% extruded) [22, 23, 25, 26]. In other work, originally from Gale et al in 1999, medial meniscal body extrusion of 3 mm or more was suggested to be “pathologic” [3, 5, 27] and is probably the most widely acknowledged cut-off for research purposes. However, to the best of our knowledge, this cut-off has not been challenged in a systematic evaluation against multiple structural pathologies of the knee joint suggestive of knee OA. Consequently, there is a lack of evidence of what may be regarded as “pathologic.”

Thus, our aims were to (1) assess the adequacy of the 3-mm cut-off to denote pathological medial meniscal extrusion and (2) determine the optimal cut-off for meniscal extrusion that would maximize the sensitivity and specificity, both with respect to other structural features of OA (as a potential consequence of meniscal extrusion), namely radiographic tibiofemoral OA, bone marrow lesions (BML), and cartilage damage.

## Material and methods

**Fig. 1:**
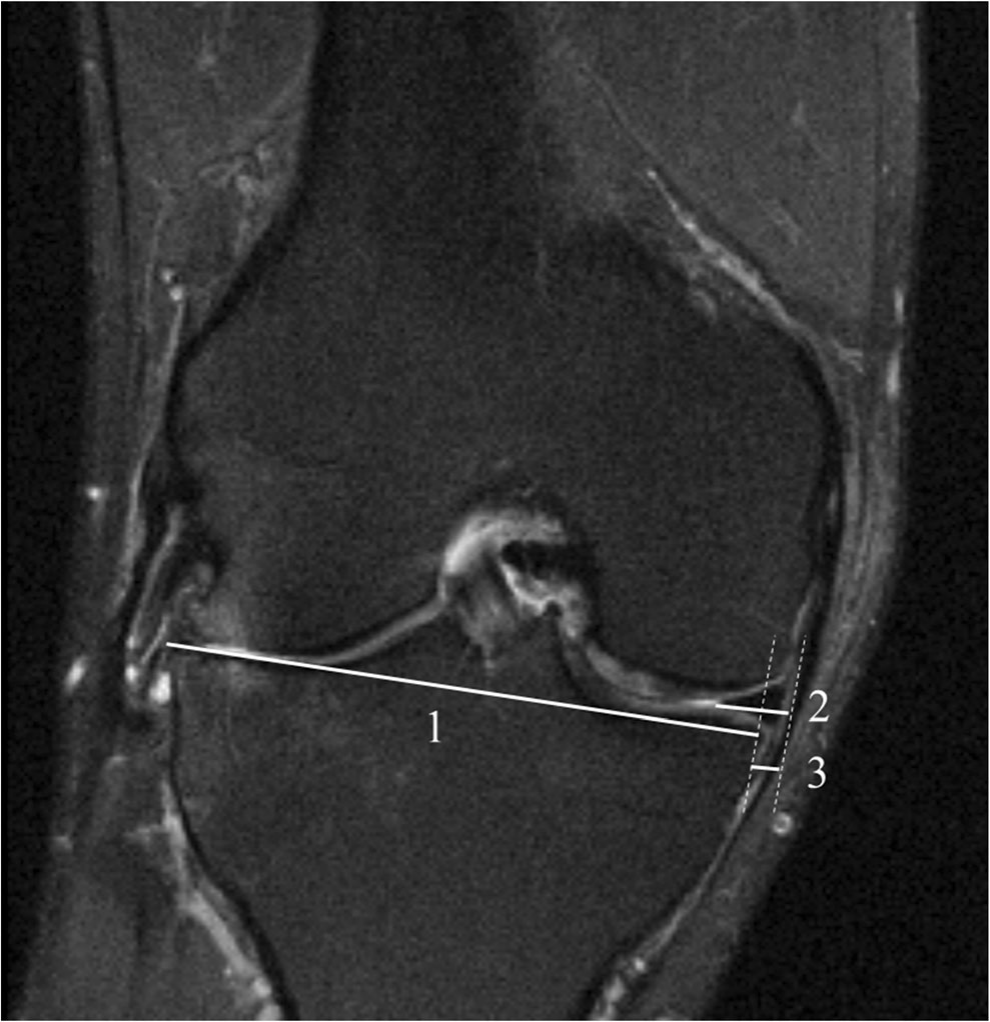
Meniscal extrusion measurements. 1 = tibia width, 2 = medial meniscal width, 3 = medial meniscal body extrusion

We used data from the well-characterized Framingham Community cohort [11, 28–30]. This cohort consists of 1039 persons from Framingham, MA, USA. The subjects were aged 50–90 years and were drawn from census tract data and random-digit telephone dialing. The selection was not made on the basis of knee or other joint problems. Subjects with a history of bilateral total knee replacement, rheumatoid arthritis, dementia, or terminal cancer and those who had contraindications to MRI were excluded. Measurement of height and weight was performed. All subjects had posteroanterior knee X-rays obtained by weight-bearing fixed-flexion protocol, and images were read according to the Kellgren and Lawrence (KL) scale [31]. The KL grading system is most commonly used for assessing severity of osteoarthritic disease in the *whole* knee joint, and we therefore made no specific discrimination for the medial compartment. MRI scans were obtained using a 1.5-Tesla scanner (Siemens Healthineers) with a phased array knee coil. We used three pulse sequences to assess meniscus position and integrity, sagittal and coronal fat-suppressed proton density–weighted turbo spin-echo (repetition time 3610 ms, echo time 40 ms, 3.5-mm slice thickness, 0-mm interslice gap, echo spacing 13.2 ms, turbo factor 7, field of view 140 mm, matrix 256 × 256) and sagittal T1-weighted spin-echo (repetition time 475 ms, echo time 24 ms, 3.5-mm slice thickness, 0-mm interslice gap, field of view 140, matrix 256 × 256). One observer (FS, an orthopedic surgeon) measured meniscal body extrusion to the nearest millimeter (mm) in the medial compartment of all knees where knee MRI was eligible for measurement of meniscus. We excluded all subjects where the MR image was unreadable or where the medial meniscal body was completely missing, i.e., no measure of meniscal extrusion could be obtained. A subset of 20 knees was re-measured by the same observer and 29 by a second reader (also an orthopedic surgeon). Both intra- and inter-reader intraclass correlation coefficient (ICC) for medial meniscal extrusion were calculated (single measures in a two-way mixed effects model). For the measurement of intra- and inter-agreement for medial meniscal extrusion measurement, the differences were visualized in the Bland-Altman plots [32]. The measurements were determined on the mid-coronal slice, where the medial tibia spine appeared largest. When it was too difficult to distinguish the maximal spine area between two or more slices, the slice with the largest tibia width was used. The point of reference for extrusion was the tibia plateau osteochondral junction at the joint margin excluding osteophytes. For the measurements, a reference line was drawn between the medial and lateral osteochondral junction, defined as the tibia width. Then, parallel to the tibia width, the medial meniscal width and meniscal body extrusion were measured. We used Merge eFilm software 3.4 and made all the measurement to the closest mm. See Fig. 1.

As described earlier [33], MRI scans were read for BMLs and cartilage damage by two musculoskeletal radiologists using the whole organ magnetic resonance imaging score (WORMS) [25]. Cartilage damage was considered present if there was a small focal loss less than 1 cm in greatest width or areas of diffuse partial or full thickness loss (WORMS grade ≥ 2 in at least one of five segments within the *medial* tibiofemoral compartment). We did not consider intrachondral signal alterations (WORMS grade 1) to represent cartilage damage. BMLs were considered present if there were non-cystic subchondral areas of ill-defined high signal on proton density–weighted MR images with fat signal suppression in the *medial* tibiofemoral compartment (WORMS grade ≥ 1 in at least one of five segments). For the X-rays, we considered knees with KL grade ≥ 2 as having radiographic tibiofemoral OA.

## Statistics

From the 1039 individuals in the cohort, 36 had missing MRI in this study. Nine hundred fifty-eight had MRI of acceptable quality and were measurable for meniscal body extrusion. For our analyses, there were 936 persons with extrusion and KL measurements, 951 with extrusion measurements and cartilage damage grades, and 953 persons with extrusion measurements and BML grades. For each outcome, radiographic OA, BML, and cartilage damage, we constructed separate receiver operating characteristic (ROC) curves and calculated the area under the curve using the medial meniscal extrusion in mm as predictor variable. The performance of the 3-mm cut-off was evaluated using all subjects in the cohort, and sensitivity, specificity, and positive and negative predictive values were calculated. We report these measures with exact binomial confidence intervals (CI). We estimated a new cut-off that maximized the Youden index [34], which combines sensitivity and specificity into a single measure (sensitivity + specificity – 1). It is the point on the ROC curve which is farthest from the line of equality and reflects the intension to maximize the correct classification rate. The performance of the new cut-off was evaluated using repeated (10 times) 10-fold cross-validation to avoid overfitting. Sampling 95% confidence intervals for sensitivity, specificity, and positive and negative predictive values were calculated [35]. We also provide the percentage of correctly classified subjects (also known as accuracy) for both cut-offs.

For data analysis and statistical software, we used Stata 14 [36] or R [37].

## Results

### Study cohort characteristics

**Fig. 2:**
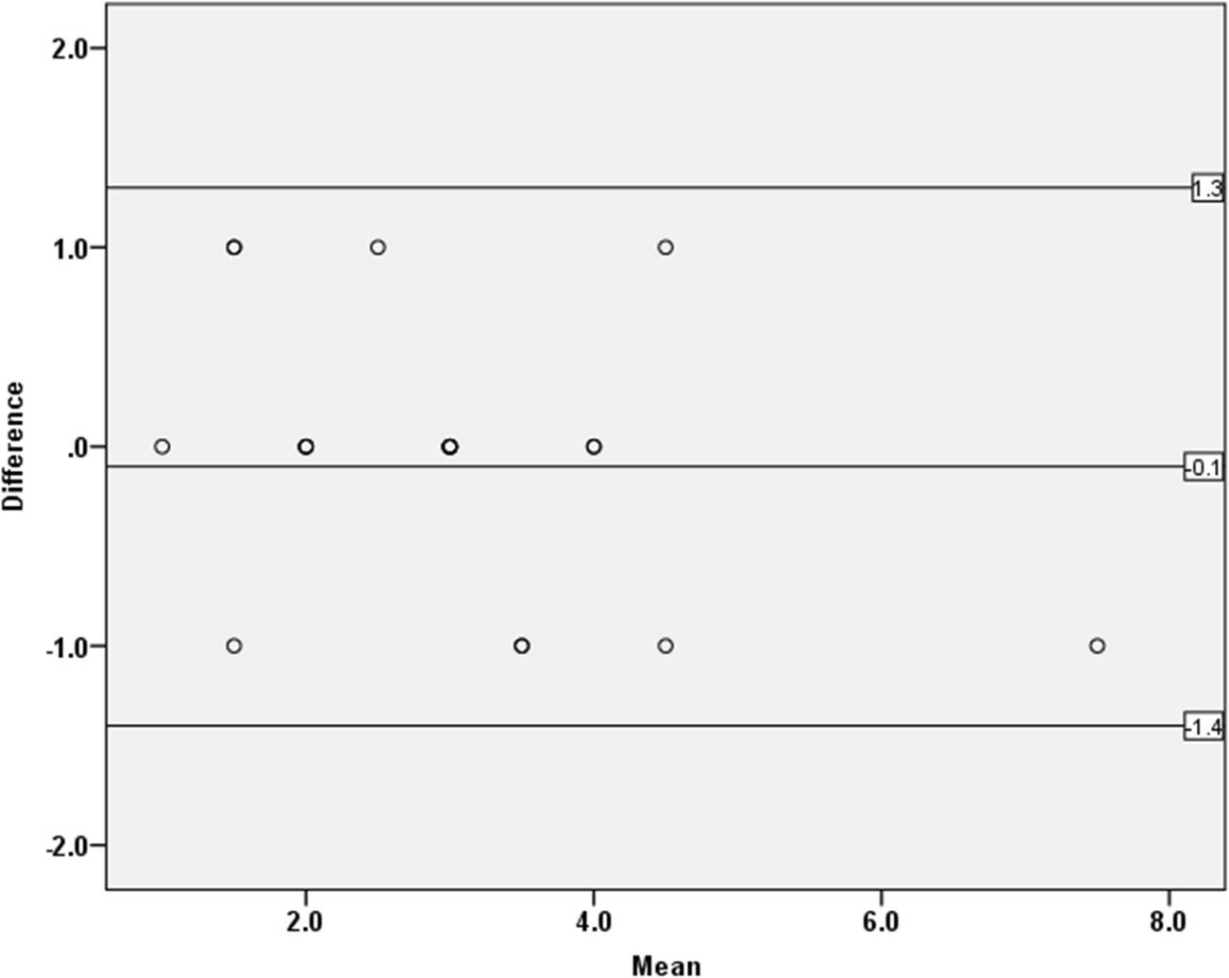
The Bland-Altman plot for intra-reader agreement of medial meniscal extrusion measurement. Upper line = upper 95% Bland-Altman confidence interval, Lower line = lower 95% Bland-Altman confidence interval, mid line = mean difference. Some dots are superimposed due to participants having the same values of both the mean and difference in medial meniscal body extrusion

**Fig. 3:**
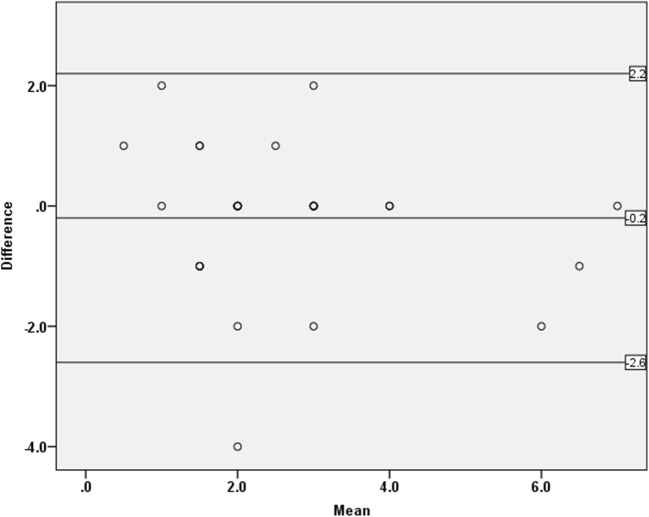
Bland-Altman plot for inter-reader agreement of medial meniscal extrusion measurement. Upper line = upper 95% Bland-Altman confidence interval, lower line = lower 95% Bland-Altman confidence interval, mid line = mean difference. Some dots are superimposed due to participants having the same values of both the mean and difference in medial meniscal body extrusion

The mean (SD) age of the included persons was 62.2 (8.5); 57.0% were women (Table 1), and the mean (SD) medial meniscal extrusion was 2.6 (1.2) mm. Compared to persons without these features, those with radiographic OA, BML, or cartilage damage had, on average, more meniscal extrusion (Table 2). The intra-reader ICC for the primary reader was 0.91 (95% CI 0.75 0.79–0.96), and the inter-reader ICC was 0.73 (95% CI 0.50–0.86). The Bland-Altman plots for intra- and inter-agreement of medial meniscal extrusion measurement are shown in Figs. 2 and 3.

**Table 1:** Descriptive statistics of the study sample. Radiographic osteoarthritis (OA) = Kellgren and Lawrence (KL) grade ≥ 2

	*N* = 958
Age, mean (SD) years	62.2 (8.5)
Sex, *n* (%)^a^	
Men	412 (43.0)
Women	565 (57.0)
Body mass index, mean (SD) kg/m^2^	28.5 (5.6)
Number (%) of knees with radiographic OA	152 (15.9)

^a^12 subjects with missing value for gender

**Table 2:** Descriptive statistics of the osteoarthritis (OA) features: radiographic OA, bone marrow lesions (BML), and cartilage damage

OA structural feature	N	Mean extrusion (mm)	Extrusion range (mm)	% with extrusion ≥ 3 mm
Radiographic OA				
No	782	2.6 (1.2)	0–9	51
Yes	154	4.5 (2.3)	0–10	81
BML				
No	685	2.6 (1.3)	0–9	49
Yes	268	3.7 (2.0)	0–10	74
Cartilage damage				
No	525	2.4 (1.1)	0–7	47
Yes	426	3.5 (2.0)	0–10	68

*N*, number of persons; *SD*, standard deviation

### Predictive ability of meniscal extrusion with respect to OA structural features

**Fig. 4:**
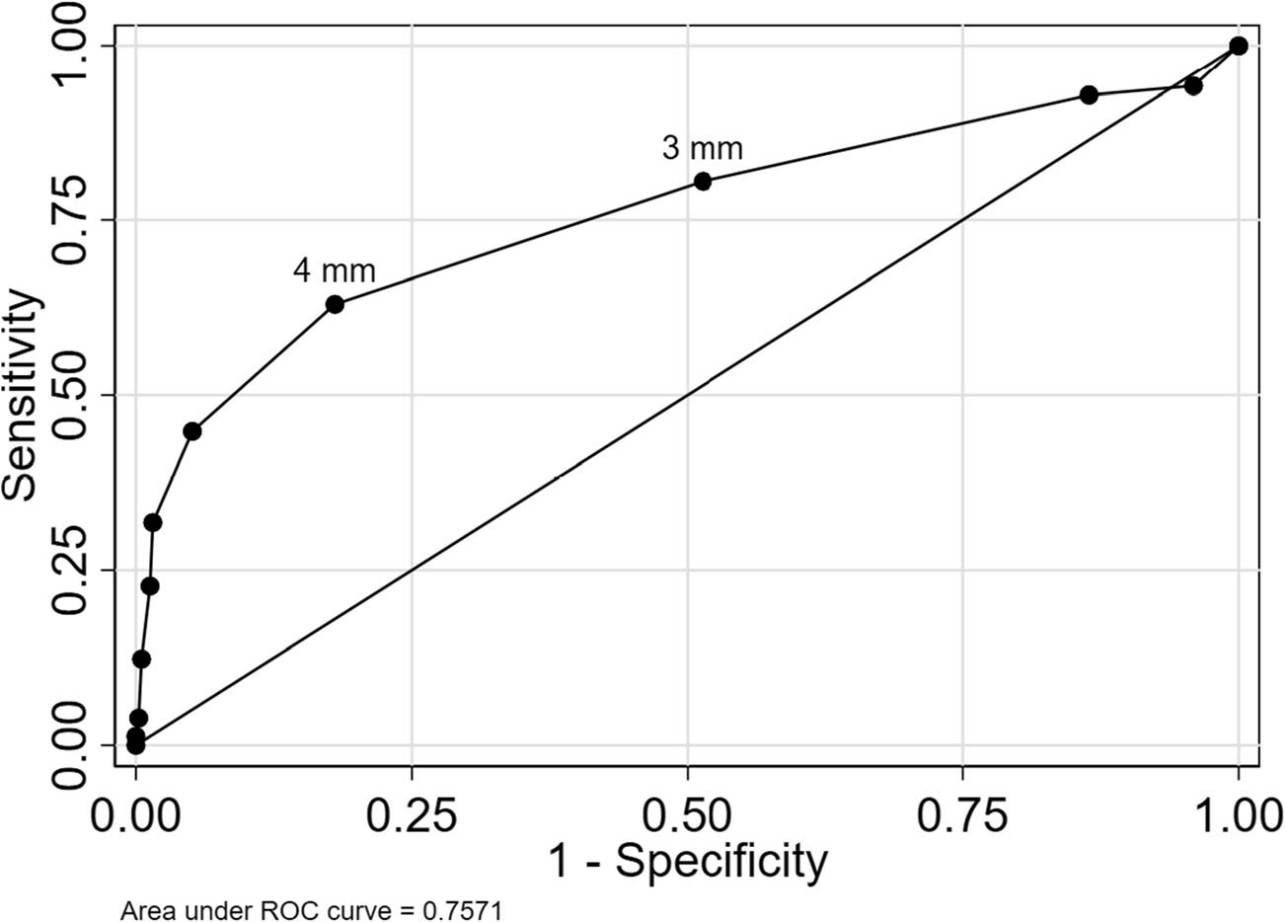
ROC curve for medial meniscal body extrusion versus radiographic OA. The cut-offs for 3 and 4 mm are pointed out

**Fig. 5:**
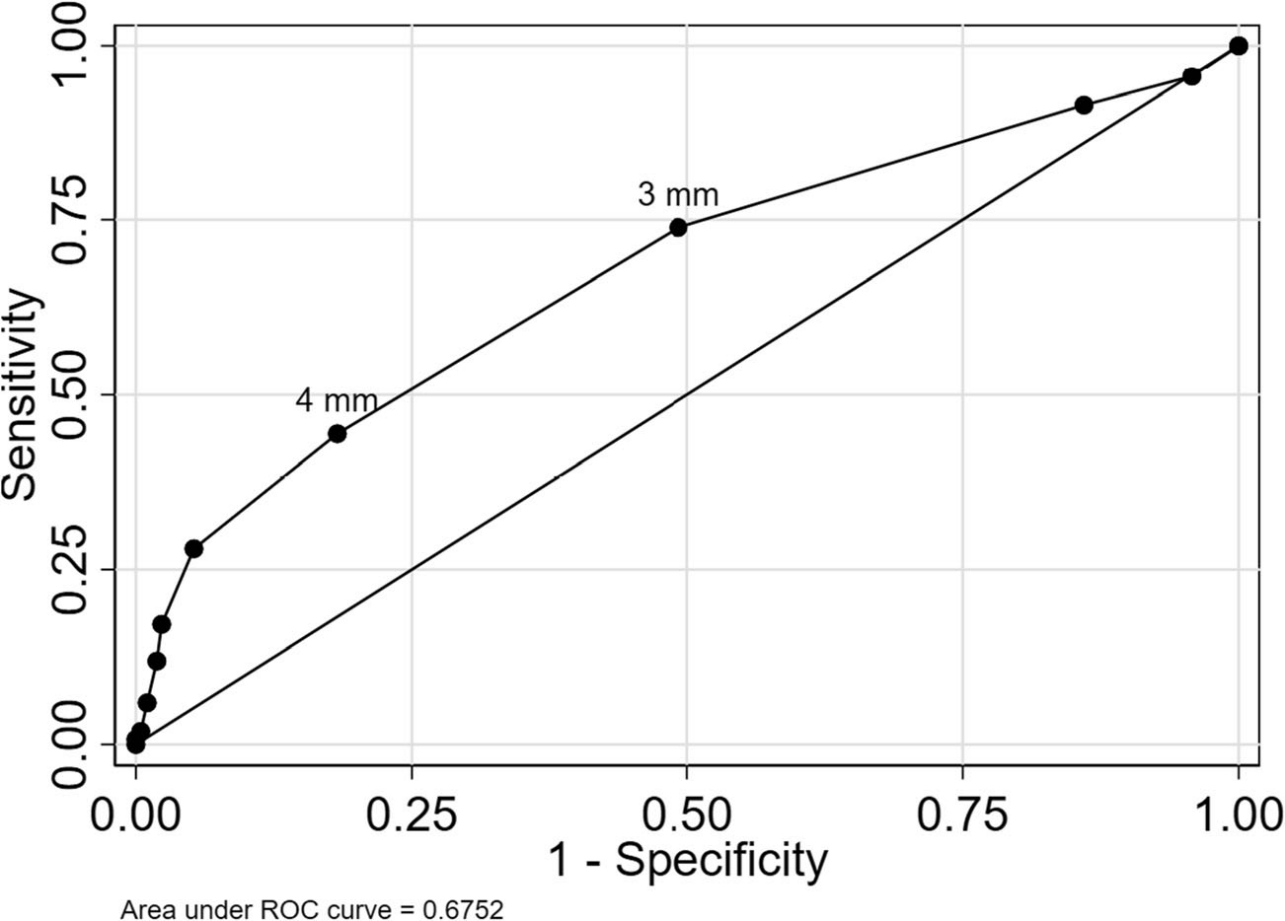
ROC curve for medial meniscal body extrusion versus BML. The cut-offs for 3 and 4 mm are pointed out

**Fig. 6:**
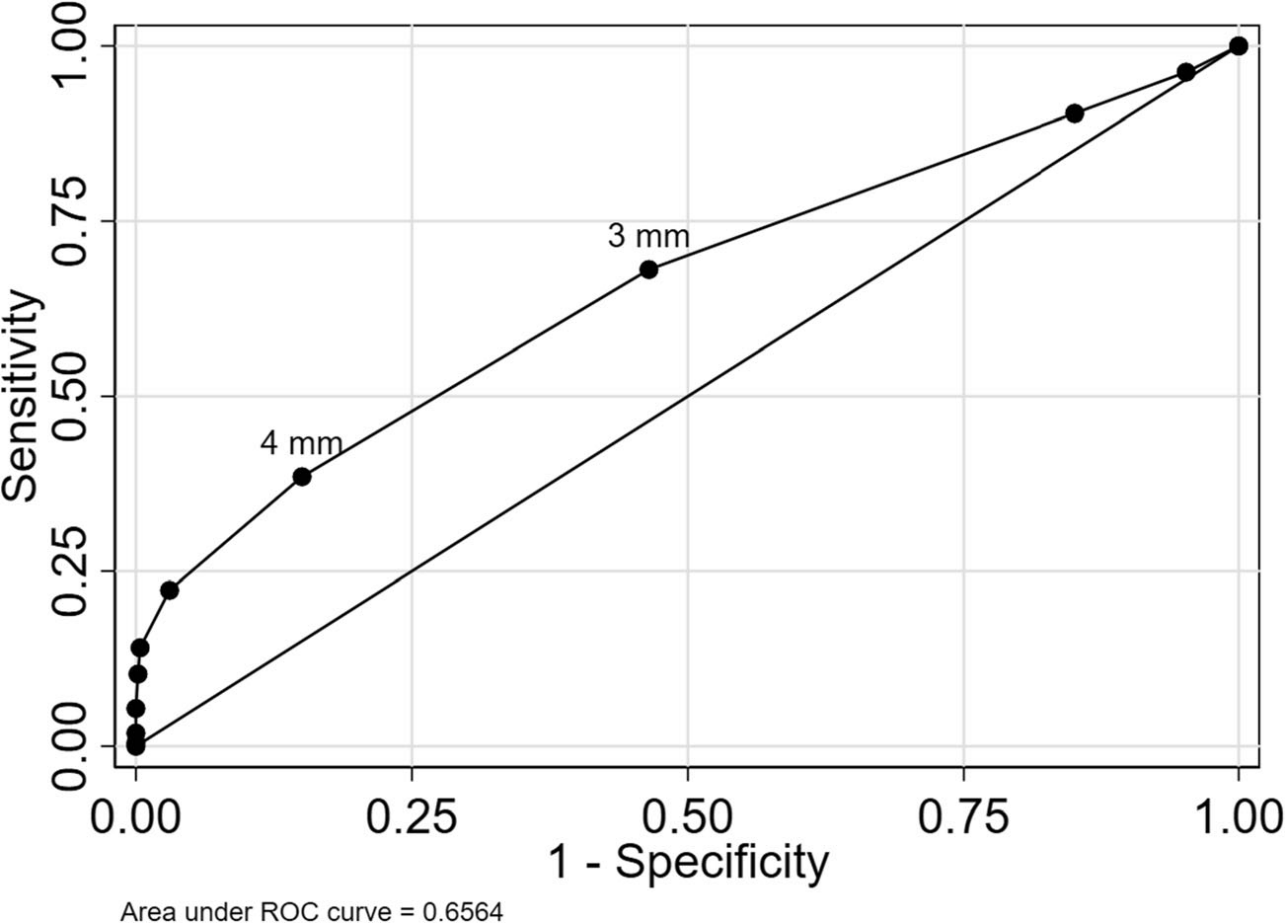
ROC curve for medial meniscal body extrusion versus cartilage damage. The cut-offs for 3 and 4 mm are pointed out

Using continuous medial meniscal extrusion as a marker of OA features yielded areas under the ROC curve of 0.76 (95% CI 0.71 to 0.81) with respect to radiographic OA, 0.67 (95% CI 0.64 to 0.71) for BML, and 0.65 (95% CI 0.62 to 0.69) for cartilage damage (Figs. 4, 5, and 6).

The commonly used cut-off for extrusion of 3 mm had sensitivity between 68 and 81% for the three evaluated outcomes, while specificity was lower in the range 49 to 54% (Table 3).

**Table 3:** Discriminatory accuracy of extrusion cut-offs

		OA structural feature	
		Radiographic OA	
Cut-off	3 mm		4 mm
Sensitivity	81 (73–87)		63 (60–65)
Specificity	49 (45–52)		82 (81–83)
Positive predictive value	24 (20–27)		41 (40–43)
Negative predictive value	93 (90–95)		92 (91–92)
% correctly classified	54 (51–57)		79 (78–80)
		BML	
Cut-off	3 mm		4 mm
Sensitivity	74 (68–79)		46 (43–48)
Specificity	51 (47–55)		79 (77–81)
Positive predictive value	37 (33–41)		48 (46–50)
Negative predictive value	83 (79–87)		79 (78–79)
% correctly classified	57 (54–61)		70 (68–71)
		Cartilage damage	
Cut-off	3 mm		4 mm
Sensitivity	68 (63–73)		39 (37–41)
Specificity	54 (49–58)		84 (82–85)
Positive predictive value	54 (50–59)		67 (65–69)
Negative predictive value	67 (63–72)		63 (62–64)
% correctly classified	60 (57–63)		64 (63–65)

Numbers are estimated as % with 95% confidence intervals in parentheses. Radiographic osteoarthritis (OA) = Kellgren and Lawrence (KL) grade ≥ 2, bone marrow lesions (BML) = WORMS grade ≥ 1, and cartilage damage = WORMS grade ≥ 2

The best cut-off maximizing the Youden index was 4 mm, with sensitivity between 39 and 63% and specificity between 79 and 82% depending on the outcome evaluated (Table 3).

## Discussion

In this study, knees with radiographic OA, BML, and cartilage damage had mean meniscal body extrusion well over 3 mm, and a large percentage had extrusion over 3 mm (for radiographic OA as much as 82%). However, while our results suggest that the 3-mm cut-off for medial meniscal body extrusion had high sensitivity, it had quite low specificity as a marker of structural OA features. Our newly estimated cut-off of 4 mm yielded higher specificity and higher proportion of correctly classified subjects. Although there are many other well-known features of OA, such as osteophytes and synovitis, we decided a priori on the three main features because they all have well-established and validated methods for image evaluation (KL grading, cartilage damage, and BMLs). In a prior study, we found that the medial compartment factors associated with meniscus position were predominantly ipsilateral meniscus tear or maceration/destruction, but the intention of the present study was to assess the medial meniscal extrusion in the knees with osteoarthritic changes [38].

We calculated a new alternative cut-off of 4 mm to suggest “pathological” medial meniscal extrusion. When analyzing this new cut-off, the main difference between the results for cut-off of 3 mm and 4 mm is a shift towards higher percentage of correctly classified subjects and a shift from high sensitivity and low specificity to lower sensitivity and high specificity. Of course, there is a “trade-off” from high sensitivity to higher specificity. This is important, not only for study purposes. Since 4-mm cut-off has a lower false positive rate, we emphasize its importance in a clinical setting. Four-mm cut-off resulted in higher percentage of correctly classified persons with respect to radiographic OA, and also BML presence and cartilage damage. This is in part a consequence of the fact that in the whole cohort, there are more persons not having the outcome than having the outcome—as expected in a cohort representative of the general population.

Our study has a number of important limitations that we would like to acknowledge. This is a cross-sectional study and therefore the 4-mm cut-off does not necessarily represent the most optimal cut-off for evaluating meniscus position as a dichotomous (yes/no) *risk factor* for the *development* of future knee OA or *worsening* of structural damage. Longitudinal datasets are needed to evaluate this cut-off. Positive and negative predictive values depend on the prevalence of the disease in the sample, which in this case (in the general population) is lower than would be expected in most clinical settings. In general, in a clinical setting (with an expected higher prevalence of structural pathologies), a higher positive predictive value and lower negative predictive value would be expected for the evaluated cut-offs. The age range of 50–90 years does not allow us to generalize our findings to younger individuals. We used a relatively simple 2-dimensional measurement technique, which does not provide as much detailed information as full segmentation of the meniscus body. The latter is however costly and time-consuming and thus often not feasible in larger study samples. The eFilm software only allowed measurements to the closest millimeter, and since meniscal extrusion differences are very small, stronger software could have been preferable. However, our measurements of meniscal extrusion had high reliability and acceptable agreement. The Framingham Community cohort is cross-sectional, but it has important strengths being population-based, i.e., representative of the general population in this age category.

In summary, this study confirms (1) that medial meniscal body extrusion is strongly associated with OA, at least for the three OA features we evaluated. (2) The cut-off value of 4 mm may be a better cut-off to use than 3 mm as it maximizes the sensitivity and specificity with respect to radiographic OA, bone marrow lesions, and cartilage damage. Thus, we suggest that medial meniscal body extrusion of 4 mm or more may be considered as an alternative cut-off to be used mainly for epidemiologic study purposes, when categorizing is necessary, and to some degree also in a clinical setting. Otherwise, using extrusion measures as a continuous variable preserves all the information and is preferable. Each cut-off results in compromising either sensitivity or specificity. We advise caution to apply any specific cut-off in a clinical setting as the association between knee *symptoms* and meniscus extrusion is still not entirely clear. This is a topic we will explore in a future study.

## Abbreviations

BLOKS: Boston-Leeds Osteoarthritis Knee Score
BML: Bone marrow lesion
CI: Confidence interval
ICC: Intraclass correlation coefficient
KL: Kellgren and Lawrence
MOAKS: MRI Osteoarthritis Knee Score
MR: Magnetic resonance
MRI: Magnetic resonance imaging
OA: Osteoarthritis
ROC: Receiver operating characteristic
SD: Standard deviation
WORMS: Whole organ MR score
